# The Influence of Irradiation Time and Layer Thickness on Elution of Triethylene Glycol Dimethacrylate from SDR® Bulk-Fill Composite

**DOI:** 10.1155/2016/3481723

**Published:** 2016-06-06

**Authors:** Ryta Łagocka, Katarzyna Jakubowska, Dariusz Chlubek, Jadwiga Buczkowska-Radlińska

**Affiliations:** ^1^Department of Conservative Dentistry, Pomeranian Medical University, Powstańców Wlkp. Avenue 72, 70-111 Szczecin, Poland; ^2^Department of Biochemistry and Medical Chemistry, Pomeranian Medical University, Powstańców Wlkp. Avenue 72, 70-111 Szczecin, Poland

## Abstract

*Objective*. This study aimed to evaluate triethylene glycol dimethacrylate (TEGDMA) elution from SDR bulk-fill composite.* Methods*. Three groups of samples were prepared, including samples polymerized in a 4 mm layer for 20 s, in a 4 mm layer for 40 s, and in a 2 mm layer for 20 s. Elution of TEGDMA into 100% ethanol, a 75% ethanol/water solution, and distilled water was studied. The TEGDMA concentration was measured using HPLC.* Results*. The TEGDMA concentration decreased in the following order: 100% ethanol > 75% ethanol > distilled water. Doubling the energy delivered to the 4 mm thick sample caused decrease (*p* < 0.05) in TEGDMA elution to distilled water. In ethanol solutions, the energy increase had no influence on TEGDMA elution. Decreasing the sample thickness resulted in decrease (*p* < 0.05) in TEGDMA elution for all the solutions.* Conclusions*. The concentration of eluted TEGDMA and the elution time were both strongly affected by the hydrophobicity of the solvent. Doubling the energy delivered to the 4 mm thick sample did not decrease the elution of TEGDMA but did decrease the amount of the monomer available to less aggressive solvents. Elution of TEGDMA was also correlated with the exposed sample surface area.* Clinical Relevance*. Decreasing the SDR layer thickness decreases TEGDMA elution.

## 1. Introduction

Resin-based composites (RBCs) play an important role in modern restorative dentistry. The longevity and safe use of these materials are influenced by both mechanical and chemical properties, as well as the dentists' technique and experience [1]. One of the most common drawbacks of all RBCs is inadequate polymerization. In clinical conditions not all of the dimethacrylate monomer is converted to a polymer and a considerable amount of residual monomer can remain in the polymerized resin composite restoration [2]. These monomers can elute from the polymerized dental methacrylate based materials [3]. The elution is a diffusion-rate-dependent process that is influenced by many parameters, such as the polymer matrix composition, surface treatment of the composite filler particles, and the nature of the solvent [2]. It has also been suggested that it is linked to the degree of curing in the polymer network [4]. Leaching of monomers can impact the structural stability and biocompatibility of the material and could at least partially account for failures observed clinically. Furthermore, the release of unreacted monomers could be responsible for undesirable biological responses, such as cytotoxicity or disturbance of pulp progenitor cells differentiation, even at nontoxic concentrations [5–11].

Recently, a new category of flowable materials called bulk-fill flowable RBCs was developed [12, 13]. The first of these launched for clinical practice was SureFil® SDR Flow (Dentsply International, York, PA). In Europe, this product was introduced in February 2011 under the name SDR (Dentsply International). The point of difference of this material is that it can be placed in 4-mm thick bulks instead of the conventional incremental placement of 2 mm thick composite layers [13–17]. The manufacturer's recommended polymerization time for the 4 mm layer is 20 s with a light intensity minimum of 500 mW/cm^2^ [14]. This light energy density is lower than that for conventional RBCs, where a light energy density of 21–24 J/cm^2^ is required for adequate photopolymerization of a 2 mm composite sample layer [18]. Compared to conventional materials, the SDR material has a different organic resin structure, doubled thickness of the polymerized composite layer, and decreased polymerization time. There are concerns about the impact of these changes on the polymerization rate and biocompatibility of this bulk-fill composite resin. Clinical studies advocate the use of bulk-fill flowable RBC bases [15, 19–21]. Investigations on clinically relevant properties of bulk-fill materials have been reported, including on marginal quality [15], cuspal deflection [19], cuspal deflection in conjunction with microleakage [20], and adhesion to cavity-bottom dentin [21]. However, there are few reports on their mechanical properties and biocompatibility. Studies on the micromechanical properties of bulk-fill flowable RBC base materials have been championed by Ilie and coworkers [12, 13]. The authors concluded that a light irradiation time of 20 s for the placement of a 4 mm bulk increment of the bulk-fill flowable RBC base material did not compromise its micromechanical properties [13]. This was corroborated by Moorthy et al. [20]. Czasch and Ilie [13] determined the degree of conversion (DC) of SDR bulk-fill samples. The mean DCs of SDR were 61 and 60% at irradiation depths of 0.01 and 2 mm, respectively. These corresponded well with results from Finan et al. [22], who obtained a mean DC of SDR of 59% at an irradiation depth of 1 mm. They observed no significant difference in the DCs for irradiation depths of 1–4 mm. However, the DCs at irradiation depths of 5–8 mm were lower and the DC decreased linearly with irradiation depth to a minimum of 45% at an irradiation depth of 8 mm [22].

Despite the fact that many studies have investigated the release of residual monomers from composite materials [2–4, 23–32], the literature regarding monomer elution from bulk-fill RBCs is limited. To date, only a few papers have been published on the release of monomers from bulk-fill flowable RBCs polymerized in 4 mm layer, with no study regarding the influence of irradiation time and layer thickness on monomer elution from these composites [33, 34]. Alshali et al. [33] study showed that elution of residual monomers from SDR bulk-fill composite resin did not differ from conventional resin composites. The study showed more powerful monomer elution from SDR to 70% ethanol solution than to the water and artificial saliva. Cebe et al. [34] study evaluated the elution of monomers from bulk-fill composite resins into 75% ethanol solution. The study showed higher concentration of the eluted TEGDMA from SDR composite than the other monomers, with the cumulative amount of eluted TEGDMA increasing with time. As mentioned above, the elution of unreacted monomers affects the biocompatibility of the composite resin. Identification and measurement of the amount of residual monomers eluted from polymers can be used to evaluate polymer quality. Leachable monomers are thought to contribute to a wide range of adverse biological reactions. Several* in vivo* and* in vitro* studies have demonstrated that the basic RBC monomers, bisphenol A glycol dimethacrylate (bis-GMA), urethane dimethacrylate (UDMA), and triethylene glycol dimethacrylate (TEGDMA) comonomer, exhibit toxic properties [5–8].

Apart from the modified basic monomers UDMA and bisphenol A ethoxylated dimethacrylate (bis-EMA), TEGDMA is the main comonomer in the SDR composite resin. Its low molecular mass makes this monomer reactive, mobile, and relatively easy to elute from the composite material matrix [2, 7]. Unreacted TEGDMA is a toxic substance exhibiting cytotoxic, genotoxic, mutagenic, and allergenic effects [5]. It exhibits systemic and local toxicity on living organisms [5–8, 35]. With regard to the clinical significance of unbound TEGDMA, it is clear that it may diffuse through dentin tubules into the pulp or elute from the restoration into the oral cavity [5, 9]. Unreacted TEGDMA monomer may be a substrate for microorganisms colonizing the marginal gap, as it can promote proliferation of cariogenic microorganisms, including* Lactobacillus acidophilus* and* Streptococcus sobrinus* [36]. The level of leaching of molecules from RBCs can be affected by several factors. According to Ferracane [2, 4], leaching is affected by the content of leachable compounds, which is in turn directly affected to the RBCs polymerization rate (e.g., energy density of delivered light, thickness of the increment). The chemistry of the solvent also has a significant effect on the elution [2]. Finally, the size and the chemical composition of the leachable species, including the polymer matrix composition, the filler particle type and content, and the porosity and homogeneity of the resin, play a role [37].

The aim of the present study was to test the following two null hypotheses: (1) the doubling of the recommended energy delivered to the 4 mm thick SDR sample would decrease the amount of unreacted TEGDMA monomer, independently of the extraction solution tested, and (2) reducing the thickness of the sample would decrease the amount of unreacted TEGDMA monomer, independently of the extraction solution tested.

## 2. Material and Methods

The flowable bulk-fill RBC SDR (Lot number 384201, Dentsply International) packaged in the form of Compula® tips was examined. According to manufacturer's information, SDR flow consists of Ba-Al-F-B-Si-glass and St-Al-F-Si-glass as fillers (68% mass fraction, 44% volume fraction) and modified UDMA, bis-EMA, and TEGDMA as the resin matrix, camphoroquinone as the photoinitiator, and the additives butylated hydroxytoluene, UV stabilizer, titanium dioxide, and iron oxides.

### 2.1. Preparation of Samples

Three groups of samples (*n* = 15 in each group) were prepared with different curing protocols (Table 1). The manufacturer's recommended polymerization time for a 4 mm material layer is 20 s. Therefore, the group of samples polymerized under these conditions was labeled as control group 4/20. The group of samples polymerized in a 4 mm layer for 40 s was labeled as group 4/40, and the group of samples polymerized in a 2 mm layer for 20 s was labeled as group 2/20 (Table 1). The samples were prepared using white Teflon moulds, which allowed the production of standardized cylindrical samples (ø 5 mm and 2 and 4 mm high). The forms were positioned on a transparent plastic strip lying on a glass plate and then filled with composite material. The samples were built up by one single increment of 2 or 4 mm (Table 1). After filling the mould with composite, a transparent plastic strip was placed on top of the composite to prevent formation of an oxygen-inhibited superficial layer. Every sample was then polymerized as detailed in Table 1. Samples were cured with a dental curing unit (G-Light, GC) with a light intensity of 1000 mW/cm^2^ and a tip diameter of 7 mm. The light was placed directly on the top of the plastic strip on top of each mould. Curing was performed only on one side of the sample to mimic clinical conditions. The light intensity of the curing light was measured using manual radiometer (Spring 2K Light Meter, SPR-SP3K, Spring Health Products, Inc., Norristown, PA). After curing, the dimensions of the samples were also measured with digital calipers (LIMIT 144550100). The composite samples with dimensions different from assumed more than 0.1 mm were rejected. The sample surface area was measured from equation *A* = 2*πr* ^2^ + *h*(2*πr*), where *A* is cylinder surface area, *r* is radius of the sample, and *h* is sample high. The samples from each group were randomly divided into three subgroups (*n* = 5 each). For each subgroup, one sample was then immediately immersed in 0.5 mL of each of the following extraction media: distilled water (Direct-Q 3 UV system, Millipore, Billerica, MA), 100% ethanol (Gradient Grade, Merck), or 75% ethanol/water solution. The samples were all placed in an Eppendorf® Thermomixer Compact at 37°C. The extraction medium was renewed after 1 h and 24 h and 3, 7, 14, 21, and 31 days. The samples were protected against light during the whole procedure. The removed extraction medium at each time was used to prepare samples for high performance liquid chromatography (HPLC).

### 2.2. HPLC Analysis

**Figure 1:**
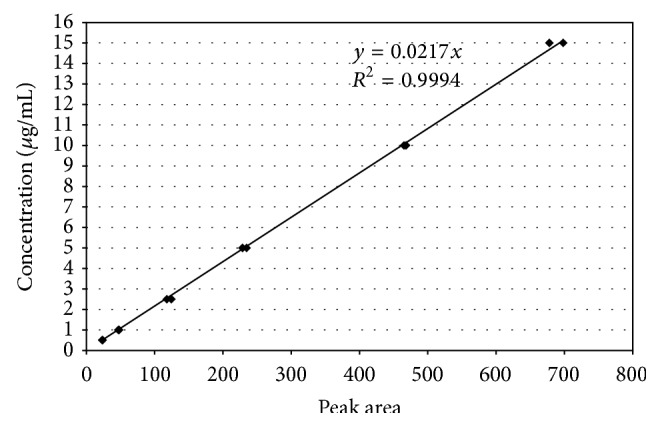
Calibration standard curve for TEGDMA monomer.

**Figure 2:**
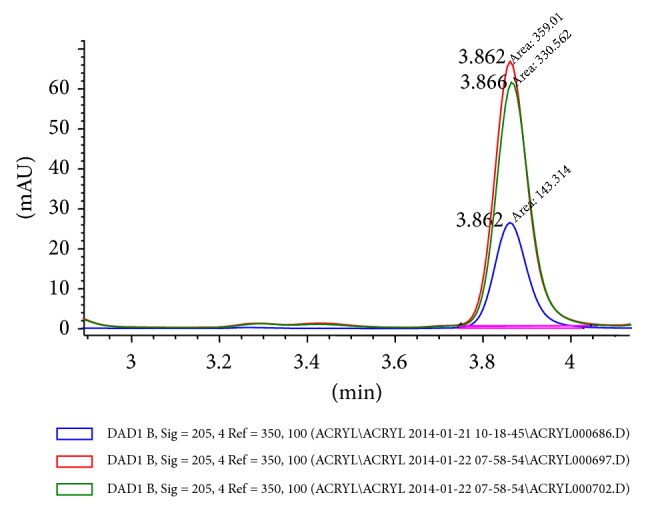
The part of the HPLC chromatograms of samples obtained after 1 day for the SDR samples from control group 4/20 in distilled water (blue), 100% ethanol (red), and 75% ethanol (green) with TEGDMA peak. The TEGDMA retention time was 3.85 min.

**Figure 3:**
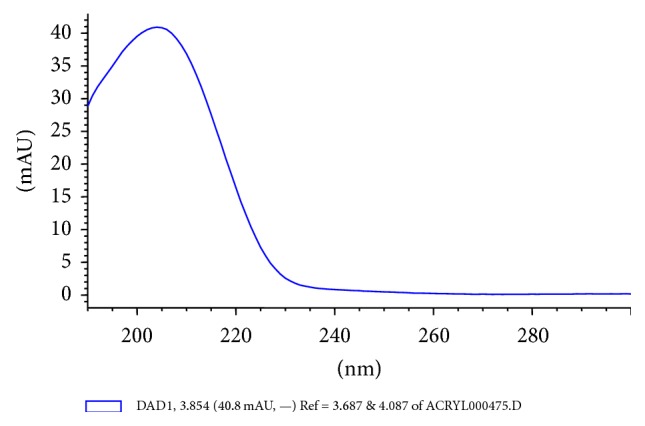
UV-Vis absorption spectrum of the TEGDMA monomer measured at 205 nm.

 HPLC analyses were performed using an Agilent Technologies (Santa Clara, CA) 1200 Series system is composed of a four-channel gradient pump (G1311A) with a vacuum degassing module (G1322A), an automated dosing system (G1367C), a DAD SL detector (61315C), and a column thermostating compartment (G1316B). Separations were conducted using a reverse phase column (LiChrospher 100 RP-18, ø 4 mm, particle ø 5 *μ*m). The column was protected using a LiChroCART 4-4 precolumn. The mobile phase was 70% acetonitrile (Gradient Grade, Merck) and 30% water (Direct-Q 3 UV system, Millipore). The mobile phase flow rate was 5 mL/min and the column temperature was 23°C. Absorbance was at 205 nm. The chromatograms were analyzed using HP Chemstation (Agilent Technologies) software. On-column injection of 10 *μ*L samples was performed every 23 min with a triple needle rinsed in 50% acetonitrile in water. All measurements were performed once for each sample. The TEGDMA monomer concentration was calculated using linear regression analysis of the results from the calibration curve. A six-point calibration curve (0.5, 1, 2.5, 5, 10, and 15 *μ*g/mL) for the TEGDMA monomer (CAS# 109-16-0, Lot# STBC5193V, Sigma-Aldrich, St. Louis, MO) was constructed using the external standard method (Figure 1). The TEGDMA retention time was 3.85 min (Figure 2). The limit of quantification was about 0.5 *μ*g/mL. Concentrations below this level could not be quantified. Identification and quantitative analysis of TEGDMA monomer in the analyzed samples was performed by comparing the elution time and the integrated UV absorption peak area of the eluates with that of the reference compound (Figure 3).

### 2.3. Statistical Analysis

Statistical analysis was performed using STATISTICA for Windows 9.0 (StatSoft, Inc.). To evaluate differences between values, the following nonparametric tests were used: Friedman's ANOVA, Wilcoxon's matched-pair test, ANOVA Kruskal-Wallis, and *U* Mann-Whitney. A probability of less than 0.05 was considered significant, and below 0.01 was considered highly significant.

## 3. Results

**Figure 4:**
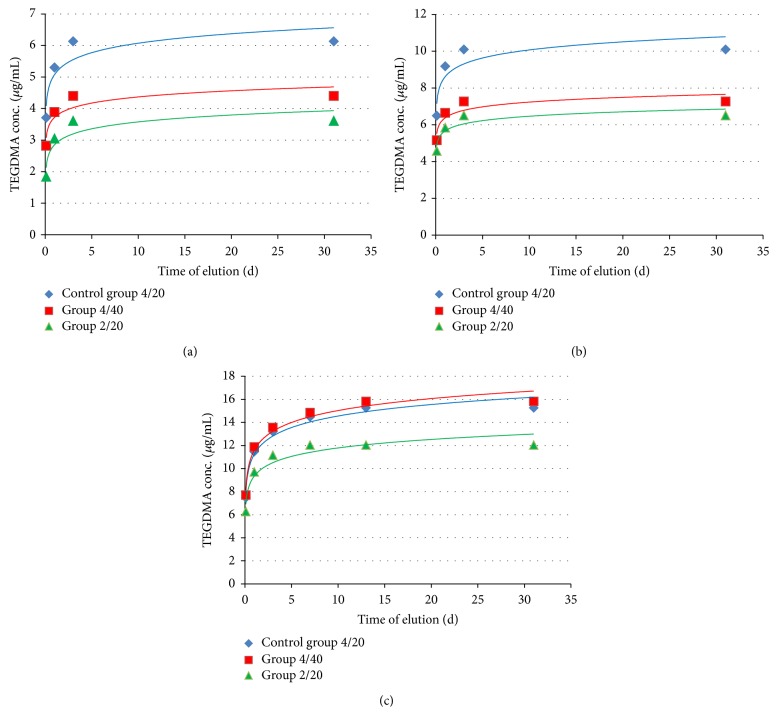
(a) The cumulative amount of TEGDMA monomer released from SDR samples to distilled water. (b) The cumulative amount of TEGDMA monomer released from SDR samples to 75% ethanol. (c) The cumulative amount of TEGDMA monomer released from SDR samples to 100% ethanol.

The mean values (*μ*g/mL) and the standard deviations for TEGDMA release are given in Table 2. Release profiles of cumulative TEGDMA concentration over time for all groups are presented in Figures 4(a), 4(b), and 4(c). All curves showed a good logarithmic fit. Sixty minutes after the polymerization, the TEGDMA monomer was detected in all the solutions (Table 2). Regardless of the curing protocol or extraction solution, the highest concentration of TEGDMA monomer was found after 1 h after photopolymerization (Table 2). The TEGDMA concentration then decreased with time. The cumulative TEGDMA concentration in the eluate after 24 h was 75–92% of the total leached monomer after 31 days (Table 2). Significant (*p* < 0.05) decreases in the monomer concentration with elution time were observed for every group. TEGDMA was eluting for the longest time in 100% ethanol. In this extraction medium, the monomer was still detected at 14 days for all groups except group 2/20, where the monomer was also present after 14 days but at a level under the method's quantification limit. In distilled water and 75% ethanol, the TEGDMA monomer was still detected on the third day of the study. In 75% ethanol, TEGDMA monomer was still present after 7 days of elution, but the level was under the method's quantification limit. The TEGDMA monomer was not detected in any solution after 21 and 31 days. For all the groups, the concentration of eluted TEGDMA monomer was the highest in 100% ethanol, followed by 75% ethanol, and then distilled water. The differences in eluted monomer concentrations among the solvents were statistically significant (*p* < 0.05) for all the groups.

Increasing the energy delivered to the 4 mm thick SDR samples (group 4/40 versus control group 4/20) influenced TEGDMA monomer elution. This effect was dependent on the immersion medium used. In distilled water and 75% ethanol, extending irradiation time from 20 to 40 s decreased TEGDMA elution from the SDR samples (Figures 4(a) and 4(b)). However this was statistically significant (*p* < 0.05) for every elution period tested only for the samples immersed in distilled water (Table 2). Also the total elution of the monomer showed statistically significant (*p* < 0.05) differences between group 4/40 and control group 4/20 only for samples immersed in distilled water (Table 2). It is worth noting that doubling the polymerization time (doubling the delivered energy) had no influence on monomer elution from SDR samples immersed in 100% ethanol (Figure 4(c)).

Decreasing the SDR layer thickness from 4 to 2 mm (control group 4/20 versus group 2/20) reduced TEGDMA monomer elution significantly (*p* < 0.05) for all solvents used (Table 2). For the samples immersed in distilled water, decreasing the sample thickness (group 2/20), compared to control group 4/20, reduced monomer elution more than doubling the energy delivered to the composite material did (group 4/40) (Figure 4(a)). The cumulative TEGDMA elution after 24 h and the entire study period was significantly lower (*p* < 0.05) for samples from group 2/20 than those from group 4/40. For samples immersed in 75% ethanol both decreasing sample thickness and extending the irradiation time reduced monomer elution to a similar extent for control group 4/20, but the differences between groups 4/40 and 2/20 were not statistically significant (Figure 4(b), Table 2). With 100% ethanol, only reducing the sample thickness decreased monomer elution (Figure 4(c)). The differences between group 2/20 and both control group 4/20 and group 4/40 were statistically significant (*p* < 0.05) for every elution time tested (Table 2).

## 4. Discussion

The results of this study showed that the time of elution and concentration of TEGDMA eluted from SDR RBC was dependent on the extraction medium used. The degree of monomer elution is proportional to the hydrophobicity and swelling capacity of the organic solvent used [2, 26, 27]. Because of their structures, TEGDMA, bis-GMA, UDMA, and bis-EMA are all soluble in the ethanol [27]. Their elution will be affected by their size, with smaller molecules, like TEGDMA, showing enhanced mobility and eluting faster than larger, bulkier molecules (e.g., bis-GMA). For the tested solvents, 100% ethanol could penetrate the resin matrix much easier than 75% ethanol or pure water, increasing sorption, swelling and plasticization, and expanding the spaces between the polymer chains [27, 38]. This facilitates elution of unbound substances from the surface and bulk of the RBC. Even though a pure ethanol extraction medium is not relevant clinically, it may allow evaluation of the amount of all alcohol-soluble leachable compounds contained in RBCs because it is more powerful solvent than 75% ethanol [30]. The monomer concentration leached into this solvent should be close to the total leachable content of unreacted monomer in the resin. The selection of an extraction medium for toxicity testing of dental resins depends on the research purpose. Because different extraction media have been used in different studies, some contradictory results have been obtained in cytotoxicity assays, for example, for TEGDMA monomer release into ethanol, compared with distilled water, and saliva [26, 27, 38, 39]. According to ISO specifications, distilled water should be used as the extraction medium for resin-based filling materials because it is representative of the humid, intraoral environment that contains both saliva and water [40]. Moharamzadeh et al. [26] found that TEGDMA release into distilled water was not significantly different to that into other water-based extraction media that could be used to simulate an intraoral environment (e.g., saline solution, artificial saliva, and Dulbecco's Modified Eagle Medium without serum). Ferracane [2] found that fluids in the oral cavity exhibit extraction behavior that lies between organic solvents and water. Therefore, the US Food and Drug Administration recommends the use of a 75% ethanol/water solution to simulate oral cavity conditions. This solvent mixture has been used in many studies [23–25, 41]. The present study was designed to investigate the elution concentration range that could be obtained with 100% ethanol (maximum elution of monomer), distilled water, and an ethanol/water mixture, which may be more clinically relevant.

To date, no study has defined the time period necessary for total elution of unreacted TEGDMA monomer from RBCs. Most studies have looked at release after 1 week or 1 month [23–25, 28]. Only a few studies have investigated long-term release. Mazzaoui et al. [42] looked at release over 3 months, Örtengren et al. [43] over 6 months, and Polydorou et al. [23] over 12 months. Because of slow diffusion of the extraction medium into the cross-linked resin matrix, it can take weeks or months for resin to become saturated with the substances. However, the monomer elution process itself seems to be complete within days, because changes in the weight of the resin after this time are very small and not measurable. As demonstrated in the present study, both the TEGDMA monomer concentration and the elution time were affected by the solvent used for elution. The highest TEGDMA monomer concentration and the longest time during which TEGDMA elution was observed (14 days) were obtained with 100% ethanol. By comparison, the monomer elution observed with 75% ethanol and distilled water was shorter (3 days). Some studies have looked at very short-term release of monomers from composite resins [27, 44]. These studies established that the maximum release of pure monomers from composite resins takes place within the first 24 h after polymerization. Nathanson et al. [44] found that the maximum TEGDMA release occurred within the first 4 minutes. Ferracane and Condon [27] report that 50% of monomers are eluted from material during the first 3 h after polymerization and 85–100% of monomers are eluted within 24 h. More recent studies using HPLC have shown that monomer elution continues beyond 24 h for RBCs [23]. However, despite further potential monomer elution, the majority of soluble substances are extracted from the material within hours. This was confirmed in the present study. Regardless of the type of extraction medium used, the majority of the TEGDMA monomer was released within the first few hours after polymerization. Monomer release exhibited a logarithmic-like profile with most of the leaching occurring in the first hours after polymerization for all the solvents tested. Our observations are similar to what is usually observed for commercial and experimental resins and resin composites [23, 24, 45, 46].

The majority of the initial elution is probably caused by release of unreacted monomers from the sample surface. After release of monomers from the surface, a slower and longer release of monomer from the bulk occurs [46]. This would imply the polymerized RBC has a heterogeneous structure. Polymerized dimethylacrylates can contain polymer areas of both low and high crosslink densities. Heterogeneity occurs almost from the beginning of RBC polymerization with the formation of highly crosslinked microgels in the suspension of unreacted monomers. The final network structure is formed by agglomeration of the microgels into clusters and then connection of the clusters [47]. Unreacted monomers may be trapped in the microgels, either between polymer chains or in spaces between polymer clusters, and this forms a monomer pool. The cross-linked polymers are practically insoluble, but they are able to swell in appropriate extraction media. The solvent penetrates into the matrix extending the spaces between the polymer chains. If a monomer is soluble in a given extraction medium, it can be eluted from the material. Monomers trapped in micropores are more susceptible to elution than those located inside the microgel [48]. Therefore, we suggest that the initial quick release occurs because of leaching from the sample surface, which is rich in organic matrix, and leaching from micropores. Leaching from the vitrified clusters occurs on a much longer time scale, for example, over 2 weeks as observed in the present study. Micropore volume is higher in more heterogeneous material. Thus, the ability to elute monomers from the material depends on not only the concentration of unreacted monomers but also on the structure of the resins and the location of monomers within the polymer network. Therefore, the concentration of TEGDMA monomer that leaches from the resin depends on the morphology of the material (e.g., composite cement, fissure sealant, bonding agent, and filling material) and its chemical structure.

The chemical structure of the resin varies with the type of monomer and comonomer used, the interactions between the monomer and comonomer, the amount of inorganic filler, and the polymerization conditions [23, 24, 32]. The organic matrix of SDR composite contains UDMA, modified UDMA, TEGDMAm and Bis-EMA [14, 47]. Compared to composites that contain bis-GMA, this matrix composition is supposed to be less viscous because these dimethacrylates form more flexible polymers [49]. High TEGDMA monomer with flexible side groups content in SDR material can decrease its viscosity too [14, 50, 51]. The SDR composite matrix contains a polymerization modulator incorporated in a high molecular weight urethane-based methacrylate resin. It is able to delay gelation and reduce polymerization shrinkage without affecting the degree of conversion [14]. Due to the conformational flexibility around the centered modulator impart, it can optimize flexibility and relax the network structure of the SDR resin. Lower shrinkage stress of SDR compared to regular flowable and nonflowable nano- and microhybrid RBCs was confirmed in a study by Ilie and Hickel [12]. Because of the polymerization shrinkage and decreased viscosity in SDR composite resin, it is thought to form a more homogenous copolymer network than conventional RBCs [12, 51, 52]. The more homogenous network could contribute to the decreased TEGDMA elution to less hydrophobic solvents.

To minimize elution of residual monomer, RBCs have to be polymerized to a high degree. The characteristics of the light source, such as energy density and spectral flux, affect the final polymerization rate [28, 53, 54]. Energy density (J/cm^2^) is the product of light intensity (mW/cm^2^) and irradiation time (s). Some studies suggested that energy density is the main determining factor in degree of conversion and mechanical properties of the composite resin [18, 55, 56]. More recent studies have reported that although radiant exposure plays an important role, light intensity, irradiation time, photoinitiator type, and filler content also influence polymer chain length, extent of cross-linking, and mechanical properties of the resin [57–60]. According to the manufacturer instructions for SDR RBC, a 20 s polymerization time is sufficient to achieve a satisfactory polymerization of a 4 mm thick composite layer [14]. Therefore, the same exposure must be sufficient for doubling the thickness of the layer in comparison to conventional resin composites. Ilie and Hickel [12] and Czasch and Ilie [13] confirmed that irradiation of 4 mm SDR for 20 s did not compromise its micromechanical properties. Finan et al. [22] obtained a mean DC of SDR of 59% at an irradiation depth of 1 mm with no significant difference in the DCs for irradiation depths of 1–4 mm. Bucuta and Ilie [61] found that bulk-fill RBCs are more translucent to blue light than conventional RBCs. For SDR one strategy to increase the depth of cure involves increasing the filler size and decreasing the filler volume fraction (44%) compared to other flowable materials [12]. Consequently, the specific surface area between the filler and organic matrix is lowered, and this reduces light scattering [61]. According to Bucuta and Ilie [61], SDR showed an increase in translucency during irradiation. In this material, 95% of the maximum irradiance was reached after 14 s of irradiation for a 4 mm thick layer [61]. Ilie and Stark [62] found that 20 and 40 s irradiation times had the same impact on the micromechanical properties of a 4 mm thick layer. Therefore, additional irradiation has no effect on the mechanical properties. Czasch and Ilie [13] found no improvement when placing thinner bulks than 4 mm or when increasing the irradiation time from 20 to 40 s for measurement depths of up to 4 mm for SDR RBC. In the present study, contradictory results were obtained for the effect of the polymerization time on elution in the different solvents. Elution of TEGDMA from bulk samples immersed in 100% ethanol was not dependent on the energy during polymerization. For this solvent, no difference was found between the 20 s and 40 s polymerization times for the 4 mm thick sample for all storage periods tested. Consequently, the first null hypothesis must be rejected. By contrast, increasing the curing time from 20 to 40 s (i.e., doubling the energy) decreased elution of TEGDMA from the 4 mm thick samples immersed in distilled water and 75% ethanol. Results for the 100% ethanol solution samples indicated that increasing the polymerization time did not affect the total amount of monomer leached from the 4 mm thick RBC layer. However, doubling the energy delivered to the 4 mm thick composite sample affected accessibility of the unbound monomer and made elution into less aggressive solvents more difficult. Several authors have investigated the correlation between the degree of conversion and amount of leachable monomer [3, 24, 63]. Tanaka et al. [63] found that increasing the irradiation time from 30 s to 50 s resulted in a significant decrease in the residual monomer contents in water. Durner et al. [3] and Sideridou and Achilias [24] determined that commercial composites showed a reduction in TEGDMA release to 75% ethanol with increased curing time. By contrast, Ferracane [2] found a very poor correlation between the degree of conversion and amount of leachable monomer. Although 40 s is commonly used as a polymerization time and thought to provide satisfactory results for the mechanical properties of composite resin, Polydorou et al. [23] concluded that this time was not more effective than a 20 s polymerization time for reducing release of monomers to 75% ethanol. Increasing the polymerization time to 80 s also did not reduce the elution.

Reducing the thickness of the composite layer greatly reduced elution of TEGDMA monomer into all the extraction media tested. This confirms the second null hypothesis. Decreasing the SDR sample thickness to 2 mm decreased the sample surface area, and this minimized elution of TEGDMA monomer. Some other studies have also found that the release depends on the exposed surface area [64, 65]. Pelka et al. [29] observed significantly more release of TEGDMA in pulverized composite samples. A meta-analysis performed by Van Landuyt et al. [30] confirmed that there was a weak but significant correlation between the exposed surface area of the tested sample and release. This was also found in the present study. The RBC samples in group 2/20 had smaller surface areas than the samples in control group 4/20 and group 4/40. A plausible physical explanation for the correlation between the release and the surface area may be that the released components mostly originate from the surface of the samples, while unbound compounds inside the polymer sample are much harder to access.

## 5. Conclusions

Both the amount of unreacted TEGDMA monomer eluted from SDR bulk-fill composite and the elution time were correlated with the hydrophobicity of the solvent used for extraction.

The results obtained with 100% ethanol revealed that doubling the energy delivered to the 4 mm composite layer did not decrease elution of the TEGDMA monomer. However, it did decrease the elution of unreacted TEGDMA monomer in less aggressive solvents.

Elution of TEGDMA monomer was also correlated with the exposed sample surface area. Decreasing the composite layer thickness decreased monomer elution independently of the solvent used.

The opportunity to decrease TEGDMA monomer elution from SDR RBC by increasing radiant exposure or decreasing layer thickness appears to be a clinically profitable method, especially in deep cavities with extensive exposed dentin surface area.

The results of this study cannot be unreservedly extrapolated across other brands of bulk-fill composites resins because of the differences in composite's matrix composition, chemistry, and the polymerization conditions. Further research on the influence of irradiation time and layer thickness on other monomers elution from different bulk-fill RBCs needs to be considered.

## Figures and Tables

**Table 1:** Description of curing protocols used for the SDR samples.

Polymerization conditions	*I*_irr_ (mW/cm^2^)	*t*_irr_ (s)	Radiant energy (J/cm^2^)	Sample thickness (mm)	Sample surface (mm^2^)
Control group 4/20	1000	20	20	4	102.05
Group 4/40	1000	40	40	4	102.05
Group 2/20	1000	20	20	2	70.65

*I*_irr_: light curing unit intensity; *t*_irr_: time of irradiation.

**Table 2:** Mean TEGDMA release (*µ*g/mL) from three groups of SDR composite resin samples measured after 1 h and 1, 3, 7, and 14 days. Between 14–21 and 21–31 days no more TEGDMA monomer was detected in any sample. The cumulative TEGDMA concentrations after 31 days are also shown. Means with same superscript symbol do not differ significantly. The other means show statistically significant (*p* < 0.05) differences.

Group	Extraction solution	Immersion time					Cumulative TEGDMA conc. after 31 d (*µ*g/mL)
		1 h	1 h–24 h	1–3 d	3–7 d	7–14 d	
		TEGDMA (*µ*g/mL) mean (SD) *n* = 5					
Control 4/20	100% ethanol	7.46 (0.20)^A^	4.00 (0.16)^B^	1.74 (0.08)^C^	1.28 (0.06)^D^	0.78 (0.02)^E^	15.26 (0.52)^F^
	75% ethanol	6.49 (1.39)^G^	2.69 (0.16)	0.91 (0.03)	bl	—	10.09 (1.58)^H^
	Distilled water	3.71 (0.28)	1.59 (0.22)	0.83 (0.12)	—	—	6.13 (0.62)

4/40	100% ethanol	7.70 (0.48)^A^	4.16 (0.17)^B^	1.69 (0.05)^C^	1.30 (0.13)^D^	0.97 (0.04)^E^	15.82 (0.69)^F^
	75% ethanol	5.16 (0.43)^G,I^	1.49 (0.07)^J^	0.61 (0.03)^K^	bl	—	7.26 (0.53)^H,L^
	Distilled water	2.82 (0.32)	1.07 (0.04)	0.51 (0.01)^M^	—	—	4.4 (0.37)

2/20	100% ethanol	6.26 (0.44)	3.45 (0.10)	1.44 (0.14)	0.88 (0.02)	bl	12.03 (0.49)
	75% ethanol	4.58 (0.17)^I^	1.26 (0.02)^J^	0.67 (0.02)^K^	bl	—	6.51 (0.21)^L^
	Distilled water	1.84 (0.05)	1.21 (0.01)	0.56 (0.03)^M^	—	—	3.61 (0.09)

—: not detected; bl: below quantification limit.
